# A Novel Insight Into the Challenges of Diagnosing Degenerative Cervical Myelopathy Using Web-Based Symptom Checkers

**DOI:** 10.2196/10868

**Published:** 2019-01-11

**Authors:** Benjamin Marshall Davies, Colin Fraser Munro, Mark RN Kotter

**Affiliations:** 1 Academic Neurosurgery Unit Department of Clinical Neurosciences University of Cambridge Cambridge United Kingdom; 2 School of Clinical Medicine University of Cambridge Cambridge United Kingdom; 3 Anne McLaren Laboratory for Regenerative Medicine Wellcome–Medical Research Council Cambridge Stem Cell Institute University of Cambridge Cambridge United Kingdom

**Keywords:** cord compression, degenerative cervical myelopathy, diagnosis, differential, spondylosis

## Abstract

**Background:**

Degenerative cervical myelopathy (DCM) is a common debilitating condition resulting from degeneration of the cervical spine. While decompressive surgery can halt disease progression, existing spinal cord damage is often permanent, leaving patients with lifelong disability. Early surgery improves the likelihood of recovery, yet the average time from the onset of symptoms to correct diagnosis is over 2 years. The majority of delays occur initially, before and within primary care, mainly due to a lack of recognition. Symptom checkers are widely used by patients before medical consultation and can be useful for preliminary triage and diagnosis. Lack of recognition of DCM by symptom checkers may contribute to the delay in diagnosis.

**Objective:**

The aims of this study were to investigate whether Web-based symptom checkers were able to recognize relevant symptoms of DCM, to characterize the DCM differential they returned , and to evaluate the diagnostic performance of recognized DCM symptoms.

**Methods:**

We pooled classical DCM symptoms from leading review articles. These symptoms were entered into the algorithms used by the top 20 symptom checker websites (N=4; Google Search). The most widely cited symptom checker, WebMD, was used to characterize the differential diagnosis for DCM symptoms.

**Results:**

A total of 31 classical DCM symptoms were identified, of which 45% (14/31) listed DCM as a differential and 10% (3/31) placed DCM in the top third of the differential. The mean differential rank for motor symptoms was significantly better than that for arthritic symptoms (*P*=.01) and the average differential rank for all symptoms (*P*=.048). The symptom checker WebMD performed best at recognizing DCM, placing the condition nearer to the top of the differential list (mean rank of 5.6) than either Healthline (rank of 12.9, *P*=.02) or Healthtools.AARP (rank of 15.5, *P*=.001). On WebMD, only one combination of symptoms resulted in DCM as the primary differential: neck, shoulder, and arm pain with hand weakness. Moreover, 151 differential diagnoses for DCM symptoms were recorded on WebMD. Multiple sclerosis and peripheral neuropathy were the most common differentials, shortlisted for 52% (16/31) and 32% (10/31) of the DCM symptoms, respectively.

**Conclusions:**

DCM symptoms are poorly identified by Web-based symptom checkers, which leads to a large differential of many other common conditions. While a diagnosis becomes more likely as the number of symptoms increases, this represents more advanced disease and will not support much-needed earlier diagnosis. Symptom checkers remain an attractive concept with potential. Further research is required to support their optimization.

## Introduction

Degenerative cervical myelopathy (DCM) is a debilitating and progressive condition that occurs when the cervical spinal cord is compressed by degenerative changes in surrounding structures. These degenerative changes, previously referred to as cervical spondylosis, include degeneration of intervertebral discs, osteophyte formation, ligamentous hypertrophy, spinal subluxation, and uncovertebral and facet joint hypertrophy [1,2].

The epidemiology of DCM is poorly characterized and has been reliant on “operative incidence” alone. This has contributed to misconceptions that it is a “rare” condition [3], whereas in fact it is estimated to be the most common spinal cord disorder [4]. For example, Kovalova et al identified that 59% (108/183) of patients from a randomly selected cohort of 40-80 year olds had magnetic resonance imaging signs of cervical cord compression and 1.1% (2/183) had undiagnosed DCM [5]. In the first prospective study of its kind, Bednarik et al showed that 8% of individuals with asymptomatic cord compression will develop DCM after 1 year and 22% over the total observation period (median follow-up, 44 months; range 2-12 years) [6]. Another more recent study has echoed these findings, with 10% of asymptomatic cord compression patients developing DCM at follow-up (median follow-up, 21 months; range 3-27 months) [7]. Given the association between DCM and age, as well as our aging population, its incidence is expected to rise.

Current treatment for DCM is limited to surgery that aims to relieve compression of the spinal cord. While most patients make a meaningful recovery, it is usually incomplete as existing damage is irreversible [8,9]. As such, treatment within 6 months of symptom onset has been shown to offer the best chance of making a full recovery [10,11].

Unfortunately, most patients wait much longer for a diagnosis; in the only study of its kind to date, Behrbalk et al found that the average time from onset of symptoms to correct diagnosis was 2.2 years [12]. Moreover, many patients go undiagnosed: in a series of neck of femur fracture patients, undiagnosed DCM was found in 18% of patients [13]. As a result, presently, most patients retain lifelong disabilities. This has a major socioeconomic impact on their lives, with unemployment, dependency, and a reduced quality of life. A recent study has demonstrated that patients with DCM have one of the lowest Short Form-36 scores among those with chronic diseases, lower than those with diabetes, cancer, chronic lung disease, and depression [14]. Thus, to improve the outcomes for such patients, an early diagnosis is needed.

Patients with DCM typically enter the health care system via primary care, and it is this interplay, between the onset of symptoms, patient presentation to primary care, consultation(s), and onward specialty referral, that makes up the majority of diagnostic delays [12]. The factors driving missed and delayed diagnosis are poorly characterized at present and difficult to investigate. However, the problem is likely multifactorial, including nonspecific and subtle early features often occurring in isolation initially, which may overlap with other conditions; incomplete neurological assessments by professionals; and poor awareness of the disease [1]. For example, in the Netherlands, a general practitioner is consulted 7 times a week for neck or upper extremity complaints (possible symptoms of DCM) of various causes [15], so distinguishing DCM can be difficult. In addition, the aforementioned Behrbalk et al series identified that 43% of patients with DCM were diagnosed and sometimes treated for carpal tunnel syndrome initially [12].

Web-based symptom checkers are websites that allow patients to select or enter a number of symptoms and using proprietary diagnostic algorithms produce a list of potential diagnoses, usually ranked in order of likelihood. These are popular with the general public; the leading engine, WebMD, receives 22 million unique visitors a month [16]. They are also frequently used prior to medical consultations; 45% of patients attending a genitourinary clinic, 47% of patients presenting to a rheumatology clinic, 53.5% of patients enrolled in a primary care practice, 52% of orthopedic outpatients, 51% of gastroenterology outpatients, 24% of adults accompanying children to a pediatric orthopedics clinic, 29% of patients referred to a medical genetics clinic, and 18% of otolaryngology outpatients with internet access had used the internet to research their symptoms prior to consultation [17-24]. Consequently, it is likely that symptom checkers are consulted when symptoms of DCM appear.

The results obtained from such websites may guide further searches and information seeking of patients. This may in turn influence their narration of symptoms and aid or obstruct diagnosis when they are seen in primary care.

This study therefore sought to investigate the recognition of DCM in Web-based symptom checkers.

## Methods

### Reported Degenerative Cervical Myelopathy Symptoms

DCM symptoms were compiled from 4 leading review articles (cited by an average of 25 PubMed Central articles) published since 2000, taken from journals spanning a range of medical expertise: rehabilitation [25], neurology [2], neurosurgery [26], and primary care [27]. Reported symptoms were extracted from the articles and consolidated into a single list by removing duplicates or overlapping symptoms. In this article, these symptoms are referred to as “classical” symptoms.

**Figure 1:**
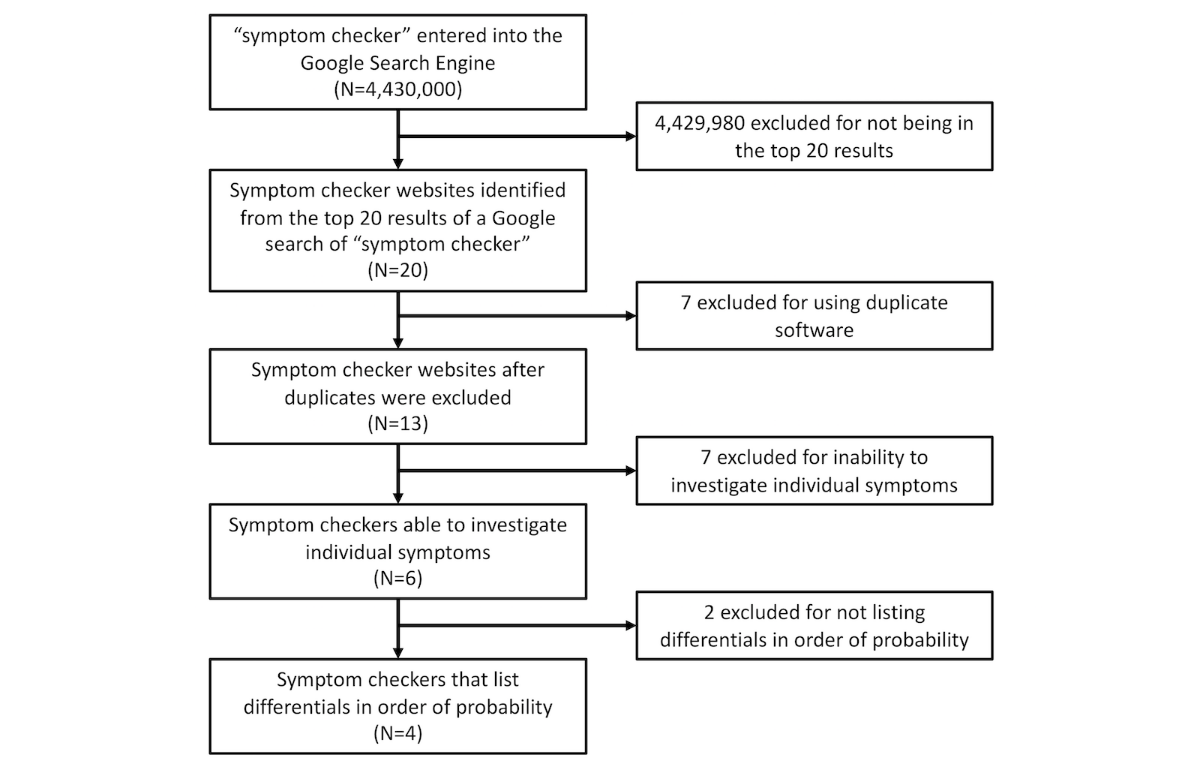
Preferred Reporting Items for Systematic Reviews and Meta-Analyses flow diagram illustrating the selection of symptom checkers.

### Web-Based Symptom Checkers

From a Google Search of “Symptom Checker” (returning over 4 million results), we selected the unique symptom checker search engines powering the top 20 symptom checkers. Checkers unable to make a diagnosis of DCM based upon symptoms or which did not list differential results in the order of likelihood were excluded (Figure 1). Consequently, we included the symptom checkers WebMD, Healthline, Healthtools.AARP, and NetDoctor. The estimated monthly visits for these websites were as follows: WebMD, 22 million [16]; Healthline, 11 million [28]; Healthtools.AARP, 3 million [29]; and NetDoctor, 1 million [30]. These symptom checker websites all described DCM under the umbrella term of (cervical) cord stenosis; however, for the purposes of this article, the term DCM will continue to be used.

Each literature-recognized DCM symptom was entered, either directly (NetDoctor) or by selecting the best match from a list of options (WebMD, Healthline, and Healthtools.AARP), into each symptom checker. When demographic information was required (WebMD and NetDoctor), the average age (57 years) and gender (male) from a recent DCM study were used [14]. If a region was required (NetDoctor), Western Europe was entered.

### Analysis

The results of the symptom checkers were combined for analysis. Metrics of performance included (1) *differential rank* for DCM (the position of DCM in a list of differentials, eg, fifth); (2) *total number of differentials* returned*;* and (3) *mean percentile rank* for DCM (percentage of conditions that were ranked below DCM in the differential list).

Additionally, WebMD, as the most widely cited and visited symptom checker (4 of the top 20 symptom checkers are powered by WebMD) with the best condition match for DCM, was selected to investigate the specificity of different combinations of symptoms and the overall differential for DCM.

To identify the most specific combination of symptoms, the symptoms that had yielded DCM as a differential were searched again in paired combinations using 2×2 probability tables, such that every combination was assessed. Symptom combinations that improved their mean ranking were carried forward into further 2×2 probability tables until no more improvements in differential rank could be achieved.

To identify the overall differential for DCM, we recorded the entire list of differential conditions for each search symptom. The number of times each of these conditions was a differential for any DCM symptom was recorded to allow a frequency chart to be plotted and the overall differential identified.

### Statistics

The Shapiro-Wilk test was used to assess for parametric distribution of data sets. The Mann-Whitney *U* test was then used to compare the means of nonparametric distributions, while a two-tailed *t* test was used to compare the means of parametric distributions. The Pearson correlation coefficient was used to assess relationships between mean differential ranks, mean percentile ranks, and mean number of differentials.

## Results

From the 4 review articles, 31 unique DCM symptoms were identified. These were grouped into motor, sensory, autonomic, and arthritic categories (Table 1) based on the domains of common outcome assessments [10]. Only abnormal gait, Lhermitte’s sign, and urinary incontinence were listed by all 4 of the articles.

Accordingly, 45% (14/31) of the symptoms entered into the Web-based symptom checkers listed DCM as a possible diagnosis. No individual symptom placed DCM as a differential in all 4 Web-based symptom checkers or as the primary differential in any one (Table 1). Of the 3 ubiquitous DCM symptoms from the literature, abnormal gait and Lhermitte’s sign did not yield DCM as a differential in the Web-based symptom checkers, and urinary incontinence had a mean rank of 15 out of 23 (percentile rank, 35). Of the symptoms which returned a differential of DCM, 14% (2/14) ranked DCM in the bottom third of differentials (percentile rank, 0-33.3), 64% (9/14) in the middle third of differentials (percentile rank, 33.3-66.6), and 21% (3/14) in the top third of differentials (percentile rank, 66.6-100). Upper limb or arm paresthesia, upper limb or arm pain (brachialgia), and hand paresthesia (with percentile ranks of 71.4, 66.7, and 70.6, respectively) were the only symptoms placing DCM in the top third of the differentials (Table 1).

**Figure 2:**
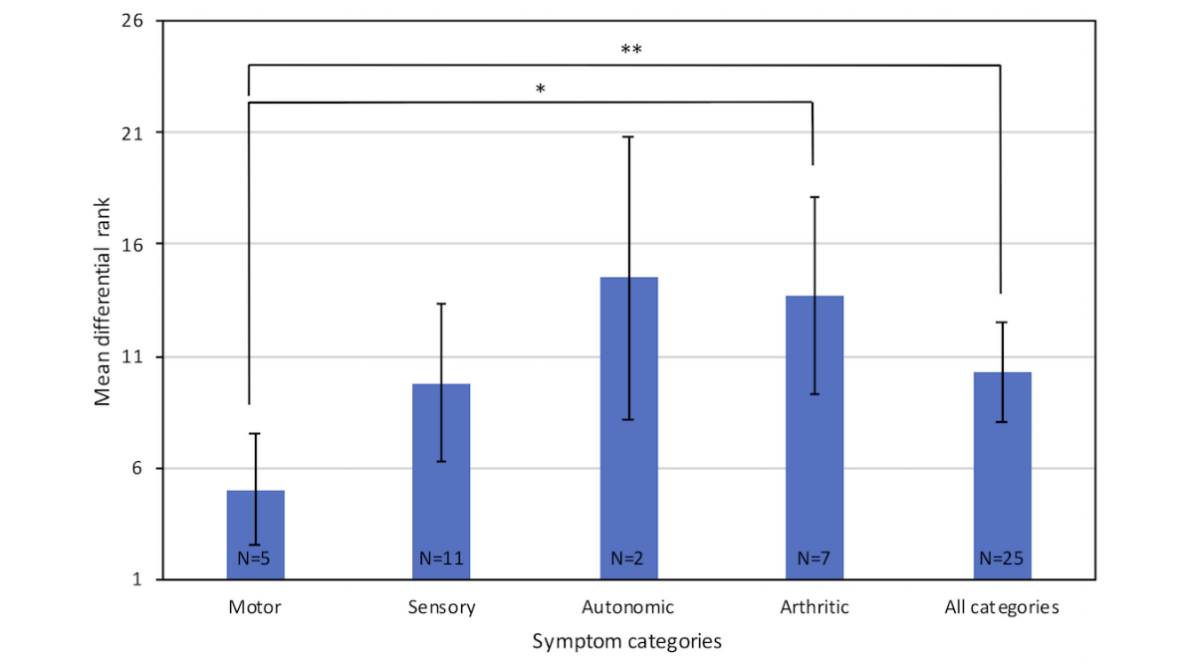
The mean differential rank for all individual degenerative cervical myelopathy symptoms, as well as the mean differential rank of the individual symptoms grouped in the motor, sensory, autonomic, and arthritic categories. Asterisk denotes a statistically significant difference between motor and arthritic categories (*P*=.01); double asterisk denotes a statistically significant difference between motor and all categories (*P*=.048).

The mean differential rank for all symptoms entered individually was 10.3, while that for the separate symptom categories were as follows: motor symptoms, 5.0; sensory symptoms, 9.8; autonomic symptoms, 14.5; and arthritic symptoms, 13.7 (Figure 2). A value of 1 would represent DCM as the top differential. The error bars indicate 95% CI. The N numbers for each category are indicated at the bottom of their respective bars.

The mean differential rank for motor symptoms was significantly better than that for arthritic symptoms (*P*=.01) and the average differential rank for all symptoms (*P*=.048). There were no other significant differences between the different symptom categories for the differential ranks, the number of differentials per symptom, or the percentile ranks.

Out of all the symptom checkers, WebMD placed DCM nearer to the top of the differential list (mean rank of 5.6) than either Healthline (rank of 12.9, *P*=.02) or Healthtools.AARP (rank of 15.5, *P*=.001). WebMD also returned fewer differential conditions for DCM symptoms (14.5) than Healthline (29.5, *P*=.01) and Healthtools.AARP (19.8, *P*=.0496). Unfortunately, comparisons with NetDoctor were not possible due to the low N number.

When symptoms were combined, on WebMD, the differential rank for DCM improved in the majority of circumstances. However, only a combination of neck pain, shoulder pain, upper limb or arm pain (brachialgia), and hand weakness placed DCM as the primary differential. There were 5 pairs of symptoms that gave DCM as the second differential and were as follows: (1) upper limb or arm weakness (paresis) and hand weakness; (2) upper limb or arm weakness (paresis) and hand numbness or sensory loss; (3) upper limb or arm weakness (paresis) and hand paresthesia; (4) hand weakness and hand numbness or sensory loss; (5) hand weakness and hand paresthesia.

For each of these pairs of symptoms, the condition ahead of DCM in the differential list was always peripheral neuropathy.

**Figure 3:**
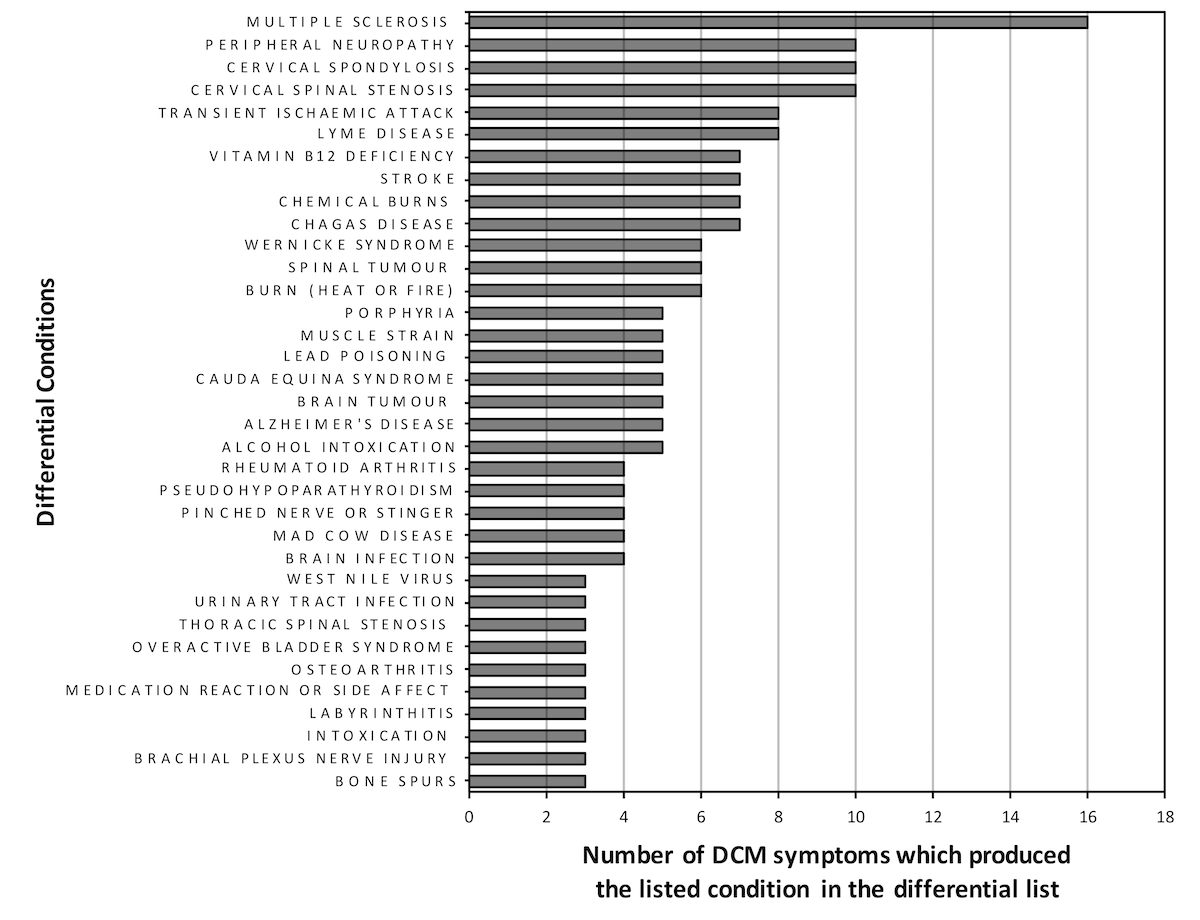
The number of degenerative cervical myelopathy (DCM) symptoms which produced the listed conditions in the differential list on WebMD.

Overall, WebMD listed 151 differentials for DCM symptoms (Multimedia Appendix 1) with multiple sclerosis and peripheral neuropathy as the most common differentials (Figure 3). The list was collated by combining all the differential lists for each individual literature DCM symptom. DCM was listed for 10 symptoms and cervical spondylosis also for 10 symptoms. Carpal tunnel syndrome was only listed as a differential for DCM symptoms once (for clumsy hands).

The number of times a symptom was referenced in the literature did not differ between symptoms that identified and did not identify DCM. In fact, there was a trend between the number of times a symptom was referenced in the literature and the mean differential rank for DCM (R=0.49, *P*=.08), meaning that the symptoms referenced more often in the literature tended to rank DCM lower down the differential list.

**Table 1:** Degenerative cervical myelopathy (DCM) symptoms compiled from the 4 review articles (if DCM was given as a differential [italicized rows], the mean rank and percentile rank in the differential list was recorded).

DCM symptom^a^		Reviews in which symptom was mentioned, n (%)	Symptom checkers in which DCM was listed as a differential, n (%)	Mean differential rank for DCM	Mean total number of differentials	Mean percentile rank in differential list^b^
**Motor symptoms**						
	Abnormal gait	4 (100)	0 (0)	N/A^c^	22	N/A
	*Loss of balance*	*1 (25)*	*1 (25)*	*8*	*14*	*42.9*
	General weakness	1 (25)	0 (0)	N/A	94	N/A
	Lack of coordination	1 (25)	0 (0)	N/A	13	N/A
	*Upper limb or arm weakness (paresis)*	*2 (50)*	*1 (25)*	*4*	*10*	*60*
	Upper limb or arm spasticity	1 (25)	0 (0)	N/A	22	N/A
	Clumsy hands	3 (75)	0 (0)	N/A	17	N/A
	Loss of hand dexterity	1 (25)	0 (0)	N/A	6	N/A
	*Hand weakness*	*1 (25)*	*1 (25)*	*4*	*5*	*20*
	Lower limb or leg weakness (paresis)	3 (75)	0 (0)	N/A	14	N/A
	*Lower limb or leg spasticity*	*1 (25)*	*2 (50)*	*5*	*19.5*	*61.2*
	Lower limb or leg jerking	1 (25)	0 (0)	N/A	24	N/A
	Lower limb or leg stiffness	2 (50)	0 (0)	N/A	15	N/A
**Sensory symptoms**						
	*Upper limb or arm numbness or sensory loss*	*2 (50)*	*3 (75)*	*10*	*27.3*	*57.1*
	*Upper limb or arm paresthesia*	*2 (50)*	*1 (25)*	*4*	*14*	*71.4*
	*Upper limb or arm pain (brachialgia)*	*2 (50)*	*1 (25)*	*4*	*12*	*66.7*
	*Hand numbness or sensory loss*	*2 (50)*	*3 (75)*	*12*	*22*	*40.7*
	*Hand paresthesia*	*1 (25)*	*1 (25)*	*5*	*17*	*70.6*
	*Lower limb or leg numbness or sensory loss*	*2 (50)*	*2 (50)*	*14*	*34*	*49.9*
	Lower limb or leg paresthesia	1 (25)	0 (0)	N/A	30	N/A
	Lhermitte's sign or phenomenon	4 (100)	0 (0)	N/A	3	N/A
**Autonomic symptoms**						
	Fecal incontinence	3 (75)	0 (0)	N/A	16	N/A
	Urgency of defecation	1 (25)	0 (0)	N/A	22	N/A
	*Urinary incontinence*	*4 (100)*	*2 (50)*	*15*	*24.5*	*40.5*
	Urinary urgency	2 (50)	0 (0)	N/A	18	N/A
	Urinary frequency	1 (25)	0 (0)	N/A	28	N/A
	Urinary hesitancy	1 (25)	0 (0)	N/A	13	N/A
**Arthritic symptoms**						
	*Neck stiffness*	*2 (50)*	*1 (25)*	*7*	*12*	*41.7*
	*Neck pain*	*3 (75)*	*3 (75)*	*15*	*21.7*	*33.0*
	Neck crepitus or clicking	1 (25)	0 (0)	N/A	16	N/A
	*Shoulder pain*	*1 (25)*	*3 (75)*	*15*	*27*	*43.4*

^a^For each symptom, the number of reviews mentioning it and the number of symptom checkers that give DCM as a differential for that symptom were recorded.

^b^The mean percentile rank for DCM in the differential list represents the percentage of conditions that were ranked below DCM in the differential list (the higher the percentile rank, the more predictive the symptom). This allowed for comparison of DCM ranking among differential lists of differing lengths.

^c^N/A: not applicable.

## Discussion

### Principal Findings

Of each of the 31 DCM symptoms identified by the 4 review articles, only 45% (14/31) reported DCM as a differential in the Web-based symptom checkers. Additionally, key or prevalent literature symptoms fared no better. Of the symptoms identified by the symptom checkers, the majority (11/14, 79%) resulted in DCM being ranked in the bottom two-thirds of differentials. Multiple sclerosis and peripheral neuropathy were the most common differentials for DCM symptoms.

Therefore, based on the current classical descriptions of DCM, symptom checkers do not perform well at diagnosing the condition; moreover, if they did, DCM appeared toward the bottom of the differential list.

### Can Symptom Checkers Have a Diagnostic Role in Degenerative Cervical Myelopathy?

Various studies have assessed the accuracy of Web-based symptom checkers with regard to linking symptoms with the correct diagnosis. For example, a recent study investigated the diagnostic accuracy of 23 symptom checkers, using 45 standardized patient vignettes, covering common and uncommon conditions (26 and 19 vignettes, respectively), as well as a range of triage urgencies (15 vignettes required emergency care, 15 required nonemergency care, and 15 required self-care) [31]. Each vignette was simplified into a core set of symptoms and entered into each symptom checker by an author with no clinical training. They found that the correct diagnosis was ranked first in 34% of patient cases and that the correct diagnosis was listed within the top 3 and top 20 differentials 51% and 58% of the time, respectively. Performance varied by the urgency of condition. The correct diagnosis was listed first for 24% of emergency cases, 38% of nonemergency cases, and 40% of self-care cases. Moreover, the correct diagnosis was listed first more often for common diagnoses than for uncommon diagnoses (38% vs 28%). Additional studies focusing on WebMD found that patients using the symptom checker in a hand surgery clinic correctly guessed a diagnosis matching that of the hand surgeon 33% of the time [32] and that with ear, nose, and throat patient cases, the symptom checker was correct 16% of the time, although the correct diagnosis was listed within the differential list 70% of the time [33].

Kobayashi et al had developed a screening questionnaire based solely on symptoms for the detection of DCM and demonstrated a sensitivity of 93.5% and specificity of 67.3% [34]. There are a number of limitations in this study, including its assessment on patients attending a neurosurgical clinic wherein the pretest probability will be greater than that in primary care, with more advanced and symptomatic patients with DCM, based on current practice. To our knowledge, this screening tool has not yet been tested elsewhere and was unfortunately unable to be tested in this study due to the complex nature of several of the questions.

The diagnostic accuracy of symptom checkers in other fields and the successful development of a symptom screening tool by Kobayashi et al [34] suggest that their accuracy in DCM could improve. As proprietary tools, their algorithms could not be interrogated, but one assumes that their performance will be limited by the poorly populated diagnostic and epidemiological evidence base in DCM [1,4].

This is acceptable as supporting early diagnosis in DCM is a major research priority, particularly in primary care where the majority of diagnostic delays occur, and the growing popularity and penetrance of symptom checkers in public health-seeking behavior [17-24] suggests they are here to stay. However, the usability of symptom checkers may have some limitations. For instance, patients are required to have a reasonable level of language and computer proficiency in order to accurately input their symptoms, and the algorithms used may be less accurate in non-Western populations, where the prevalence of certain conditions may differ. Nevertheless, as single entities accessed by potential patients with some pretest probability and accessible by professionals as decision support tools, their optimization is more attractive as an intervention than standard alternatives, such as widespread education programs.

### Apparent Degenerative Cervical Myelopathy Knowledge Gaps in Symptom Checkers

When considering the performance of symptom checkers with clinical practice, a number of deficiencies or knowledge gaps were identified, which if resolved, could optimize their diagnostic performance.

The symptom checkers generated a large differential for DCM, exemplifying the nonspecific nature of the symptoms and the diagnostic challenge. Of these differentials, alongside multiple sclerosis, peripheral neuropathy was predominant. In the only study so far to consider diagnostic practice in DCM, Behrbalk et al identified that 43% of patients were initially misdiagnosed with carpal tunnel syndrome [12]. The distinction of DCM from neuropathy is clearly a common pitfall.

The focus of symptom checkers is skewed to the upper limb, with neurological dysfunction. Furthermore, pain is a key symptom for the detection of DCM, particularly upper limb pain combined with neck and shoulder pain, which was required to promote DCM to the top of the differential list in WebMD. This is an interesting finding, as pain is neither always present in DCM nor an indication for treatment [35] and in fact often not recorded in many DCM clinical trials [10,36]. It is possible that this is a result of cervical radiculopathy, of which pain is typically a feature, being included by the term “cord stenosis” on the symptoms checkers. However, conversely focusing on diagnostic practice, Mizer et al found that upper limb pain had the best diagnostic odds ratio for DCM, with a value of 29.00, out of a long list of symptoms [37]. Therefore, while pain has not been a focus for treatment research, its significance in early diagnosis requires further evaluation.

DCM also affects the lower limb and autonomic nervous system, yet these symptoms demonstrated poor diagnostic utility in symptom checkers. This is significant, as some recent work suggests that lower limb symptoms and signs may in fact be the earliest clinical presentation of DCM, with gait disturbance being the most frequently presenting symptom in a prospective study of patients with initially nonmyelopathic cervical cord compression [38].

DCM only comes to the forefront of a differential diagnosis when multiple symptoms are reported. Unfortunately, this is likely to reflect more advanced disease, as assessed by the modified Japanese Orthopaedic Association scale and signs of cord compression on magnetic resonance imaging [39-42]; further research is required for early disease detection.

### Potential Directions for Optimization of Symptom Checkers in Degenerative Cervical Myelopathy

Clearly generating the necessary epidemiological and early presentation data confounding standard clinical practice is likely to be helpful. However, this is not straightforward, and its current absence more likely reflects the difficult practicalities of conducting such research [4]. However, the lack of specificity of classical DCM symptoms may always be a limitation, and avenues to improve this would also be helpful.

An interesting finding from the aforementioned Kobayashi et al study was the predictive power of symptoms we would consider “atypical,” for example, chest tightness. This was not identified in this study as a “classical” symptom, yet in their series, the odds ratio of chest tightness in myelopathy patients compared with controls was 22.9 [34]. Other atypical symptoms reported as prevalent among patients with DCM include the following: respiratory dysfunction, with a reduction in both forced vital capacity and forced expiratory volume in 1 second in patients with DCM compared with that in controls (65% vs 88% and 72% vs 96%, respectively) [43]; hypertension (47% vs 30%, respectively) [44]; and headaches (88% of patients with DCM undergoing surgery) [45]. In these studies, all of these symptoms improved with surgical treatment of DCM, and none of these symptoms are typical of many of the identified DCM differentials. The prevalence of atypical symptoms therefore warrants more attention, as their combination with “classical” symptoms might help improve the specificity of screening tools.

Clinical examination is an important component of medical diagnosis. Moreover, it may be that symptoms alone are insufficient for screening of DCM and physical examination findings could help. This is not straightforward as, similar to symptoms, examination findings are inconsistent. Rhee et al found that although myelopathic physical signs were significantly more common in patients with DCM than in controls with neck-related complaints (79% vs 57%, respectively), the signs were not highly sensitive for diagnosing DCM, with 21% of patients with DCM having no myelopathic signs at all [46]. However, similar to symptoms, combinations of examination findings may improve diagnostic accuracy. Tejus et al showed that a combination of the finger flexion, Hoffman’s reflex, and plantar reflex could be effectively used as a marker of cervical spinal cord compression in patients with neck-related complaints, with a sensitivity of 91.7% and a specificity of 87.5%, and their absence had a negative predictive value of 77.8% [47]. While examination findings would be limited to professional use, they could be a helpful addition to diagnostic algorithms, and their predictive power with symptoms requires further assessment.

### Conclusions

Classical DCM symptoms perform poorly in Web-based symptom checkers, in particular lower limb and autonomic symptoms. While combinations of symptoms improve the diagnostic accuracy, this will not be useful for early diagnosis. With over 150 potential differentials listed, detecting DCM early is difficult. The development of accurate diagnostic strategies is needed to support earlier diagnosis and improve patient outcomes. Symptom checkers remain an attractive concept with potential. Further research in this area is required.

## Appendix

Multimedia Appendix 1 An alphabetical list of all the potential differential conditions given for literature degenerative cervical myelopathy symptoms by WebMD.

## Abbreviations

DCM: degenerative cervical myelopathy
