# Supplementary material for: A Novel Insight Into the Challenges of Diagnosing Degenerative Cervical Myelopathy Using Web-Based Symptom Checkers
Source: J Med Internet Res. 2019 Jan 11;21(1):e10868. doi: 10.2196/10868 (PMC6330198; doi:10.2196/10868)
Supplement: Multimedia Appendix 1 [file jmir_v21i1e10868_app1.pdf]

Multimedia Appendix 1. An alphabetical list of all the potential differential conditions given for literature degenerative cervical myelopathy symptoms by WebMD.

Complete list of degenerative cervical myelopathy symptom differentials from WebMD		
Acoustic neuroma	Emphysema	Pick disease
Alcohol Intoxication	Epilepsy	Pinched nerve or stinger
Alcohol withdrawal	Excessive fluid intake	Plague
Alzheimer's Disease	Exercise or physical activity	Polycystic kidney disease
Anemia	Frozen shoulder	Polymyalgia rheumatica
Anemia (chronic disease)	Gastrointestinal bleeding	Pompe disease (late onset)
Anemia (hemolytic)	Generalized anxiety disorder	Porphyria
Anemia (iron deficiency)	Giant cell arteritis (temporal arteritis)	Pre-leukemia (myelodysplastic syndrome)
Aortic regurgitation	Glycogen storage disease Type II	Progressive supranuclear palsy
Aortic stenosis	Gout	Prostate cancer
Asperger syndrome	Heart attack	Prostate gland enlargement
Aspirin Poisoning	Heart rhythm disorder	Prostatitis
Atrial fibrillation	Heat exhaustion	Pseudogout
Autism	Histoplasmosis	Pseudohypoparathyroidism
Barbiturate abuse	Hydronephrosis	Psoriatic arthritis
Benzodiazepine abuse	Hyperthyroidism	Pulmonary hypertension
Bladder cancer	Hypopituitarism	Repetitive motion injuries
Bladder outlet obstruction	Hypothyroidism (adult)	Restless legs syndrome
Bladder stones	Impingement syndrome	Rheumatoid arthritis
Bone spurs	Insulin reaction (hypoglycemia)	Rotator cuff injury
Brachial plexus nerve injury	Interstitial cystitis	Sarcoidosis
Brain aneurysm	Intoxication	Scurvy
Brain infection	Intracranial hematoma	Separated shoulder
Brain tumor	Labyrinthitis	Septic arthritis
Broken (fractured) hand	Lead poisoning	Shingles (herpes zoster)
Broken (fractured) neck vertebra	Low blood pressure (hypotension)	Sleep deprivation
Burn (heat or fire)	Low potassium (hypokalemia)	Spinal meningitis
Bursitis	Lumbar spinal stenosis	Spinal tumor
Carpal tunnel syndrome	Lung cancer (nonsmall cell)	Stroke
Cat-scratch disease	Lung cancer (small cell)	Supraventricular tachycardia
Cauda equina syndrome	Lupus	Tendinitis
Cervical herniated disk	Lyme disease	Tetanus
Cervical spinal stenosis	Mad cow disease	Tetany
Cervical spondylosis	Median nerve injury	Thalassemia
Chagas disease	Medication reaction or side effect	Thoracic spinal stenosis
Chemical burns	Meningitis	Thyroid storm
Cocaine abuse	Middle ear infection	Tick bite
Cold exposure	Migraine headache	Torticollis
Congestive heart failure	Mononucleosis	Transient ischemic attack
Coronary artery disease	Multiple sclerosis	Tuberculosis
Crohn's disease	Muscle strain	Ulcerative colitis
Cryptococcosis	Myasthenia gravis	Ulnar nerve injury
Cyanide poisoning	Neck strain	Urethritis
Degenerative disk disease	Osteoarthritis	Urinary incontinence (overflow)
Dehydration (adult)	Osteomyelitis	Urinary incontinence (stress)
Diabetes insipidus	Overactive bladder syndrome	Urinary tract infection
Diabetes Type 1	Panic attack	Vitamin B12 deficiency
Diabetes Type 2	Parkinson disease	Wernicke syndrome
Diabetic ketoacidosis	Peripheral neuropathy	West Nile virus
Diabetic neuropathy	Phlebitis	Whiplash
Dislocated shoulder		
